# Investigating Eating Behaviors and Symptoms of Oral Frailty Using Questionnaires

**DOI:** 10.3390/dj7030066

**Published:** 2019-06-29

**Authors:** Tsukasa Hihara, Takaharu Goto, Tetsuo Ichikawa

**Affiliations:** Department of Prosthodontics and Oral Rehabilitation, Tokushima University, Graduate School of Biomedical Sciences, 3-18-15, Kuramoto, Tokushima 770-8504, Japan

**Keywords:** chewing, eating behaviors, oral frailty, oral function, questionnaire survey

## Abstract

A questionnaire survey was conducted to investigate eating behavior and the subjective symptoms of oral frailty, and to examine the relationship between them. A total of 744 subjects with ages over 65 years were included. The questionnaire comprised 18 question items indicating eating behavior and seven question items indicating oral frailty. All items were assessed according to 4 grades on a scale of 1 (not applicable) to 4 (applicable). The total score of oral frailty gradually increased with age. Regarding the scores for “eating recognition” and “eating habits”, no changes were observed, however the scores for “eating action” demonstrated a decreasing tendency with age and the scores of ≥ 85 years age group was significantly lower than the 65–69, 70–74, and 75–79 years age groups. As a result of multiple regression analysis, among the significant independent variable, the scores of “I do not chew foods well” under the category of “eating action” showed the highest standard partial regression coefficients for dependent variable of symptoms of oral frailty. The significant association was found between the eating behavior and subjective symptoms of oral frailty, and this study suggests that the good chewing habit might be an important criterion for the prevention of oral frailty.

## 1. Introduction

The elderly population as well as elderly people requiring care in Japan has remarkably increased [1,2,3]. In addition, the cost of medical and nursing care is increasing and has become a critical national issue [4]. Thus, countermeasures and prevention strategies with regard to the need for nursing care are required. The concept of frailty, which indicates the loss of physiological reserves that increases the risk of disability, has been proposed and has received increasing attention in recent years [5,6]. Sarcopenia, defined as an age-related decline in lean body mass, muscle mass, and function, is a similar concept [7]. Moreover, the importance of “taking meal”, including nutrition management and food intake, has been emphasized to prevent frailty and sarcopenia in the elderly, which is also helpful in preventing metabolic syndrome. We assessed the importance of “taking meal”, especially focusing on individual “eating behaviors” and reported the relationship between eating behaviors and body mass index in subjects from the 30s to 50s age group [8]. However, the relationship in the elderly has not been reported.

In dentistry, the concept of oral frailty, associated with the decline in oral function, was proposed in 2015 after the concept of frailty was widely introduced in Japan [9,10]. Oral frailty is considered to indicate one of the earliest stages of physical frailty. It refers to a mild decline in oral function, with symptoms such as the decline in tongue action, spilling foods, and slight choking. We previously performed a questionnaire survey and reported subjective symptoms focusing on oral frailty and physical frailty in subjects from the 40s to 90s age group [11]. However, the association between subjective symptoms of oral frailty and eating behaviors, mentioned above, in the elderly has not been investigated. It is unclear how oral functions, such as mastication and swallowing, are related to eating behavior.

In this study, a questionnaire survey was conducted to investigate eating behavior and subjective symptoms of oral frailty in elderly people and examine the association between the two.

## 2. Methods

### 2.1. Study Population and Survey

Overall, 744 subjects with ages > 65 years (257 males and 487 females, mean age 74.3 ± 7.1 years) were included in the analysis. Patients were from the Tokushima University Hospital dental division and 10 private clinics in Tokushima. Participants in academic or public classes on health, and professional staff or their families from nursing homes in Tokushima and Fukuoka were included. This questionnaire survey was executed using two methods. The collective method which is of collecting the responses after subjects gathered in a certain place, was applied for patients and participants in academic or public classes on health; and the detention method which is of leaving questionnaire sheets in each place and picking up them after a certain period, for professional staff or their families from nursing homes. All subjects who could understand the questionnaire details and answer each question by themselves were enrolled. The participants provided informed consent before their enrollment. The answer to the questionnaires was regarded as an approval of consent for other subjects. This study was approved by the Ethics Committee of the Tokushima University Hospital (No. 2404, Data of Approval: 28 September 2015) and conformed to the principles of the Declaration of Helsinki.

### 2.2. Questionnaire

For surveys on eating behavior and symptoms of oral frailty, the questionnaire used is presented in Table 1. For the survey on eating behavior, a Yoshimatsu-Nakamichi (YN) questionnaire was used [8]. This questionnaire comprised three categories with six questions in every category: “eating recognition”, related to the cognitive regulatory system of eating behavior such as motivation and consciousness of eating; “eating habit”, indicating the problems in dietary composition such as irregular meals and using fast-food restaurant; and “eating action”, related to the manner of dietary intake.

For assessing the symptoms of oral frailty, the questionnaire comprised seven question items indicating slight functional decline in oral function, such as mastication or swallowing [11]. These items on the symptoms of oral frailty corresponded to the concept of “deterioration of oral function” proposed by the Japanese Society of Gerodontology [12]. All items in the eating behavior and oral frailty questionnaires were assessed according to four grades on a scale of 1 (not applicable) to 4 (applicable), and higher scores represented a tendency toward worsening and functional decline. In addition, the basic characteristics of subjects such as sex and age were obtained.

### 2.3. Analysis

Age-dependent changes in the category scores of eating behavior and total scores of symptoms of oral frailty were assessed. A one-way analysis of variance with Bonferroni post hoc tests was used to compare age-dependent changes in each score. Multiple regression analysis was used to examine the age-adjusted association between eating behavior and symptoms of oral frailty. The dependent variable was total scores of symptoms of oral frailty, and the independent variable was each score of eating behavior. All statistical analyses were conducted with a significance level of 0.05 using the SPSS^®^ software (version 24.0, IBM Corp., Armonk, NY, USA).

## 3. Results

Table 2 presents the individual attributes of the age group and sex. The number of female subjects was more than that of male subjects in every age group. The highest number of subjects was observed in the 65–69 years age group for both male and female subjects.

**Figure 1:**
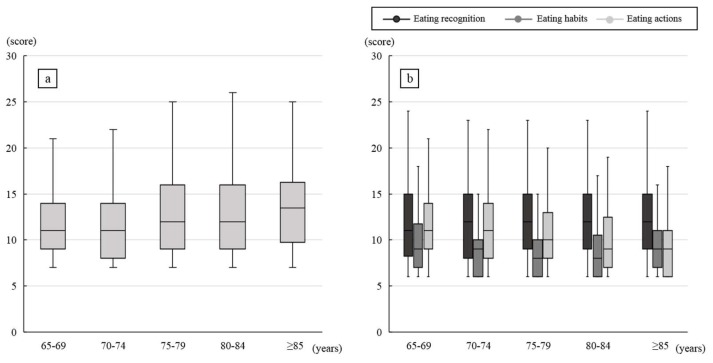
Age-dependent change in symptoms of oral frailty and each eating behaviors (**a**): symptoms of oral frailty, (**b**): eating behaviors. The box plot indicates the median with ± 75/25 quartile and minimum/maximum values.

Figure 1 illustrates the age-dependent changes in total score of oral frailty and the category scores of eating behavior. The total score of oral frailty gradually increased with age even in the elderly, and the score of ≥ 85 years age group was significantly higher than that of the 65–69 (*p* = 0.000) and 70–74 years age groups (*p* = 0.001). Regarding the scores of eating behaviors, no changes in the scores for “eating recognition” and “eating habits” were observed; however, the scores for “eating action” demonstrated an decreasing tendency with age, and the scores of the ≥ 85 years age group was significantly lower than that of the 65–69 (*p* = 0.000) and 70–74 (*p* = 0.007) age groups.

Table 3 presents the age-adjusted results of multiple regression analysis. For the dependent variable of total scores of symptoms of oral frailty, a significant independent variable was observed in one question item for “eating recognition”, (p = 0.005) two question items for “eating habit”, (*p* = 0.013, 0.039) and three question items for “eating action”, (*p* = 0.000~0.031). Among the significant independent variable, the scores for “I am speed eating” only showed negative standard partial regression coefficients (*p* = 0.031), and the scores of “I do not chew foods well” under the category of “eating action” showed the highest standard partial regression coefficients (*p* = 0.000). Regarding multicollinearity, which is a phenomenon in which one predictor variable in a multiple regression analysis can be linearly predicted from the others, no multicollinearity was noted among the independent variables, and the variance inflation factor (VIF) values ranged between 1.151 and 1.627.

## 4. Discussion

The present study attempted to clarify the association between eating behavior and subjective symptoms of oral frailty to demonstrate the decline in oral function using a questionnaire method. The eating behavior was evaluated by the YN eating behavior questionnaire, a short form of the eating behavior questionnaire proposed by Yoshimatsu, which increases awareness about behavioral problems and modifications to be made by obesity patients. Nakamichi et al. reported the reliability and validity of the YN eating behavior questionnaire and also stated that a total high score was related to one mouthful volume (bite size), which indicated obesity with high body mass index [8]. The symptoms of oral frailty were evaluated using the questionnaire with seven question items, and the efficacy of the questionnaire to screen the conditions of the elderly were reported. Each of the seven question items can be used based on the assessments of the “deterioration of oral function” according to the Japanese Society of Gerodontology. We reported that the score of oral frailty constantly increased with age, in particular, the score for, “I feel spilling foods and the difficulty to chew than before”, significantly increased in the 50s and 60s age groups. Consequently, we continued to investigate the association between eating behavior and oral frailty considering the above findings.

The scores of oral frailty gradually increased with age. Several studies on oral frailty, which focus on tooth loss and decline in oral function with age, have been performed. Heintze reported that total salivary flow at rest decreased with age in female subjects [13]. Utanohara reported that there was a difference in maximum tongue pressure between males and females in the > 60s age group [14]. Kohno reported that the occlusal force was reduced by approximately one-half in the 60s to 80s age groups [15].

Regarding the scores for “eating recognition” and “eating habits”, no specific changes were observed; however, the scores for “eating action” demonstrated a decreasing tendency with age. “Eating action”, which is the third category of eating behavior, depended on factors such as eating speed and bite size of foods. With regard to the relationship between aging and “eating action”, Kimura et al. reported a negative correlation between age and meat intake frequency [16], and Feldman et al. reported that the required time before swallowing and number of mastication increased with aging [17]. The reduction in the scores of “eating action” is supported by these reports.

The score for “I am speed eating” only showed negative standard partial regression coefficients for symptom of oral frailty. Speed eating causes an increase in the total amount of energy while ingesting foods, lack of feeling of fullness, and insulin resistance, which finally leads to obesity [18,19,20,21,22]. Considering that speed eating requires smooth and prompt actions of the jaw, lip, and tongue from food feeding to swallowing, speed eaters may have a high gnathological ability to chew speedily, thereby revealing the negative correlation of speed eating with the score of oral frailty.

The score for “I do not chew foods well” under the category of “eating action” showed the highest standard partial regression coefficients. The phrase “do not chew foods well” is easily confused with “cannot chew foods well.” The phrase “cannot chew foods well” indicates only oral frailty. However, the score of “do not chew foods well” was significantly correlated to the score of “speed eating”, and “do not chew foods well” did not mean “cannot chew foods well”. The behavior of “do not chew foods well” may lie upstream of oral frailty. Furthermore, “chewing food well” or “well-chewed food”, concerns a digestive process, and is associated with the risks of metabolic syndrome and obesity [23,24].

This study had limitations. First, there was a possibility of sampling bias that included many subjects with high health awareness patients, such as those in the university hospital and participants in academic/public classes on health. Second, a sampling bias for sex may be present because the number of female subjects was more in each age group. Third, compared with the YN questionnaire, the questionnaire for assessing the symptoms of oral frailty was not sufficiently validated. Fourth, this study was performed as a cross-sectional study.

We hypothesized that fostering appropriate eating behaviors at a young age is necessary for maintaining proper nutrition in the elderly stage. In this study, significant association between the eating behaviors of “I do not chew foods well” and symptoms of oral frailty was found in the elderly. The guidance of appropriate eating behaviors including good chewing habit of the elderly, should be noticed more to prevent oral frailty in a “super-aged” society. Future longitudinal studies to assess the change in eating behaviors and symptoms of oral frailty will be needed with more objective evaluations.

## Figures and Tables

**Table 1:** Questionnaire of symptoms of eating behaviors and oral frailty.

Questions	Estimation			
**Questionnaires on “eating recognition” of eating behaviors**				
· I feel uneasy when the there is little food left in the refrigerator.	1	2	3	4
· I keep a food around me.	1	2	3	4
· I subconsciously eat a food whenever another person eats.	1	2	3	4
· I eat a food when I have a free time.	1	2	3	4
· I eat fruits and sweets.	1	2	3	4
· I have a soft spot for sweet foods.	1	2	3	4
**Questionnaires on “eating habit” of eating behaviors**				
· I swing by a convenience store.	1	2	3	4
· I go out to eat and use delivery service.	1	2	3	4
· I have an irregular eating pattern.	1	2	3	4
· I take extra order in the restraint and delivery service.	1	2	3	4
· I use fast foods.	1	2	3	4
· I live on meat.	1	2	3	4
**Questionnaires on “eating action” of eating behaviors**				
· I am speed eating.	1	2	3	4
· I gain weight in taking a long holiday.	1	2	3	4
· I do not chew foods well.	1	2	3	4
· I regret eating too much.	1	2	3	4
· I eat with having my mouth full of foods.	1	2	3	4
· I continue to eat without a rest.	1	2	3	4
**Questionnaires on oral frailty**				
· I have dental problem than before.	1	2	3	4
· I am aware of saliva problem than before.	1	2	3	4
· I bite cheek and tongue than before.	1	2	3	4
· I drop foods while eating than before.	1	2	3	4
· I feel the difficulty to chew than before.	1	2	3	4
· I feel non smoothness of tongue actions than before.	1	2	3	4
· I am aware of swallowing action than before.	1	2	3	4

Circle the relevant number in each question. 1: not applicable; 2: occasionally; 3: sometimes; 4: applicable.

**Table 2:** Demographics of the study subjects.

Age (Years)	Male	Female	Total
65–69	98	152	250
70–74	53	103	156
75–79	43	114	157
80-84	32	75	107
≥85	31	43	56
Total	257	487	744

**Table 3:** Results of multiple regression analysis after age-adjusted. (* *p* < 0.05).

Category	Questions on Eating Behaviors	β	t-Value	VIF
Eating recognition	I feel uneasy when the there is little food left in the refrigerator.	0.065	1.899	1.160
	I keep food around me.	0.006	0.173	1.368
	I subconsciously eat a food whenever another person eats.	0.025	0.635	1.612
	I eat a food when I have s free time.	0.073	1.881	1.520
	I eat fruits and sweets.	0.058	1.457	1.610
	I have a soft spot for sweet foods.	0.101 *	2.801	1.298
Eating habit	I swing by a convenience store.	0.023	0.671	1.151
	I go out to eat and use delivery service.	0.039	1.131	1.210
	I have an irregular eating pattern	0.070 *	2.067	1.167
	I take extra order in the restraint and delivery service.	0.091 *	2.487	1.335
	I use fast foods.	0.058	1.603	1.335
	I live on meat.	0.018	0.509	1.204
Eating action	I am speed eating	−0.081 *	−2.156	1.436
	I gain weight when taking a long holiday.	0.054	1.522	1.279
	I do not chew foods well.	0.209 *	5.418	1.494
	I regret eating too much.	0.01	0.245	1.627
	I eat with having my mouth full of foods.	0.026	0.700	1.360
	I continue to eat without a rest.	0.101 *	2.692	1.403
